# Stepwise participation of HGF/MET signaling in the development of migratory muscle precursors during vertebrate evolution

**DOI:** 10.1186/s40851-018-0094-y

**Published:** 2018-06-18

**Authors:** Noritaka Adachi, Juan Pascual-Anaya, Tamami Hirai, Shinnosuke Higuchi, Shunya Kuroda, Shigeru Kuratani

**Affiliations:** 1 Laboratory for Evolutionary Morphology, RIKEN Center for Biosystems Dynamics Research (BDR), 2-2-3 Minatojima-minami, Chuo-ku, Kobe, 650-0047 Japan; 2 Laboratory for Evolutionary Morphology, RIKEN Cluster for Pioneering Research, 2-2-3 Minatojima-minami, Chuo-ku, Kobe, 650-0047 Japan; 3 Department of Biology, Graduate School of Science, Kobe University, Kobe, 657-8501 Japan; 4 Present address: Aix-Marseille Université, CNRS, IBDM UMR 7288, 13288 Marseille, France

**Keywords:** Hepatocyte growth factor HGF, Receptor tyrosine kinase MET, Hypobranchial muscles, Limb/fin muscles, Migratory muscle precursor cells, Vertebrate evolution

## Abstract

**Background:**

The skeletal musculature of gnathostomes, which is derived from embryonic somites, consists of epaxial and hypaxial portions. Some hypaxial muscles, such as tongue and limb muscles, undergo de-epithelialization and migration during development. Delamination and migration of these myoblasts, or migratory muscle precursors (MMPs), is generally thought to be regulated by hepatocyte growth factor (HGF) and receptor tyrosine kinase (MET) signaling. However, the prevalence of this mechanism and the expression patterns of the genes involved in MMP development across different vertebrate species remain elusive.

**Results:**

We performed a comparative analysis of *Hgf* and *Met* gene expression in several vertebrates, including mouse, chicken, dogfish (*Scyliorhinus torazame)*, and lamprey (*Lethenteron camtschaticum)*. While both *Hgf* and *Met* were expressed during development in the mouse tongue muscle, and in limb muscle formation in the mouse and chicken, we found no clear evidence for the involvement of HGF/MET signaling in MMP development in shark or lamprey embryos.

**Conclusions:**

Our results indicate that the expressions and functions of both *Hgf* and *Met* genes do not represent shared features of vertebrate MMPs, suggesting a stepwise participation of HGF/MET signaling in MMP development during vertebrate evolution.

**Electronic supplementary material:**

The online version of this article (10.1186/s40851-018-0094-y) contains supplementary material, which is available to authorized users.

## Background

The vertebrate skeletal musculature can be divided into two major categories: cranial muscles derived from head mesoderm, and trunk muscles that originate from somites. In gnathostomes, the trunk muscles are further divided into two groups: epaxial muscles, innervated by the dorsal ramus of the spinal nerve and located dorsal to the horizontal myoseptum, and hypaxial muscles, which are innervated by the ventral ramus of the spinal nerve and situated ventral to the myoseptum [1–4].

The epaxial muscles include muscles of the back associated with vertebrae and the occipital bone, while the hypaxial muscles comprise the subvertebral muscles, body wall muscles, hypobranchial muscles (HBMs, including the tongue muscles), and limb and fin muscles [2]. Of these hypaxial muscles, the tongue and limb muscles have attracted attention due to their unique mode of development, in which their precursors delaminate from the epithelial dermomyotome and travel long distances from their site of origin before muscle formation [5–13]. Intriguingly, the derivatives of these migratory muscle precursors (MMPs) have exhibited great morphological diversity in vertebrate history, serving as a key muscular group in helping to understand the evolution of the skeletal muscle system [2, 14–16].

Previous studies have identified a number of genes involved in regulating somitic myogenesis, including several key genes involved in MMP development [17–30]. Of these, *hepatocyte growth factor* (*Hgf*; also known as *scatter factor*) and receptor tyrosine kinase *Met* (also known as *hepatocyte growth factor receptor*, *c-Met* or *c-met proto-oncogene*) have been shown to play important roles in MMP development. Mice with mutations in these genes show severe defects in MMP derivatives, including muscles of the tongue, diaphragm and limbs, in addition to liver and placental abnormalities [17, 31–37].

Detailed analyses of embryos have suggested two major functions of HGF/MET signaling during MMP development. The first is in facilitating the delamination of the ventral dermomyotome, an initial step in MMP formation. This is supported by observations that *Met* is expressed in the ventral dermomyotome at the limb level, *Hgf* is expressed in the adjacent limb bud mesenchyme, and cells in the ventral dermomyotome do not show de-epithelialization in *Hgf* or *Met* mutant mice [17, 33, 38–41]. Implant experiments using HGF-soaked beads in chicken and zebrafish further demonstrated its role in dermomyotomal delamination [10, 42–45]. The second role of HGF/MET signaling is exerted during the migration of MMP cells. This is supported by the abnormal development of MMP derivatives in mutant mice, and the expression of *Met* in migrating limb myoblasts and *Hgf* along the migration route and at the target site, as well as the induction of myoblast translocation by the ectopic application of HGF in the limb bud [17, 33, 36, 40, 46–49].

Based on analyses of expression and function of *Hgf* and *Met* genes in mouse, chicken and zebrafish, the regulation of MMP development by the HGF/MET signaling pathway is assumed to be a shared feature in vertebrates [16, 17, 33, 45, 50]. However, most of these studies have focused on the expression and function of *Hgf* and *Met* during forelimb muscle (FLM) development, and even an expression profile of both genes is lacking in other MMP derivatives. Furthermore, analysis of *Hgf* and *Met* expression has been performed exclusively in Osteichthyan species (bony fishes), such as mouse and zebrafish, but not in chondrichthyes or cyclostomes (cartilaginous fishes and jawless fishes, respectively), representing two other major groups of vertebrates. If *Met* and *Hgf* expressions are commonly detected in the migratory myoblasts and their surrounding tissues, respectively, during the delamination and migration processes of muscles other than limbs, HGF/MET signaling would be generally linked to MMP development. In addition, if a similar expression pattern is observed for *Hgf* and *Met* in the MMPs of different vertebrate taxa, HGF/MET signaling would appear to be a shared mechanism for vertebrate MMP development. To test these possibilities, we investigated *Hgf* and *Met* expressions in mouse, chicken, shark and lamprey embryos, representing all major groups of vertebrates, by focusing on the development of HBMs, together with limb/fin muscles.

## Methods

### Sample collection

Mouse (*Mus musculus*, C57BL/6 strain), chicken (*Gallus gallus*), shark (*Scyliorhinus torazame*), and Arctic lamprey (*Lethenteron camtschaticum*) embryos were obtained as described in Adachi et al. [51], and staged according to Theiler [52], Hamburger and Hamilton [53], Ballard et al. [54], and Tahara [55], respectively.

### Molecular cloning and phylogenetic analysis

*Hgf* and *Met* genes of the shark and the lamprey were isolated by the method described in Adachi et al. [56] and [51]. The accession numbers and primer sequences used in the present study are listed in the Additional file 1: Table S1. Phylogenetic analysis of HGF and MET was performed as described in Adachi et al. [51]. Briefly, the amino acid sequences of orthologous genes in other vertebrates have been assembled from GenBank (http://www.ncbi.nlm.nih.gov/) and Ensembl (http://www.ensembl.org/index.html), and then aligned with MAFFT [57] (http://www.ebi.ac.uk/Tools/msa/mafft/). Gaps in the multiple sequence alignments were removed by trimAl version 1.2 [58] and formatted into NEXUS format by readAl v.1.2 (bundled with the trimAl package). Bayesian inference with MrBayes 3.2.6 was performed under the assumption of an LG + I + G evolutionary model [59].

### In situ hybridization

Whole-mount in situ hybridization was performed following the method described in Adachi et al. [60]. Five different probes and a slightly modified method of Adachi et al. [56] with 5% dextran sulfate in the hybridization solution, were also used for in situ hybridization of shark *Met* gene. Sections were prepared after whole-mount in situ hybridization as described in Adachi et al. [51]. Images of whole embryos were photographed with a Leica MZ16FA (Leica Camera AG, Wetzlar, Germany), and images of sections were taken with an Olympus BX53 light microscope (Olympus, Tokyo, Japan).

## Results

### *Hgf* and *Met* expression during development of the HBM and FLM in mouse embryos

We first observed the expression patterns of *Hgf* and *Met* genes in mouse embryos, in which the involvement of HGF/MET signaling in both delamination and migration of myoblasts has been demonstrated [11, 13, 17, 33, 34, 36]. Because previous studies have shown that *Hgf* is expressed in the limb bud mesenchyme and *Met* is expressed in the ventral part of dermomyotome at the limb level before the delamination and in the migrating limb myoblasts, we mainly assessed the development of HBMs from embryonic day (E) 10, when the muscles initiate delamination from anterior somites [17, 38–41, 46, 48, 61].

**Fig. 1:**
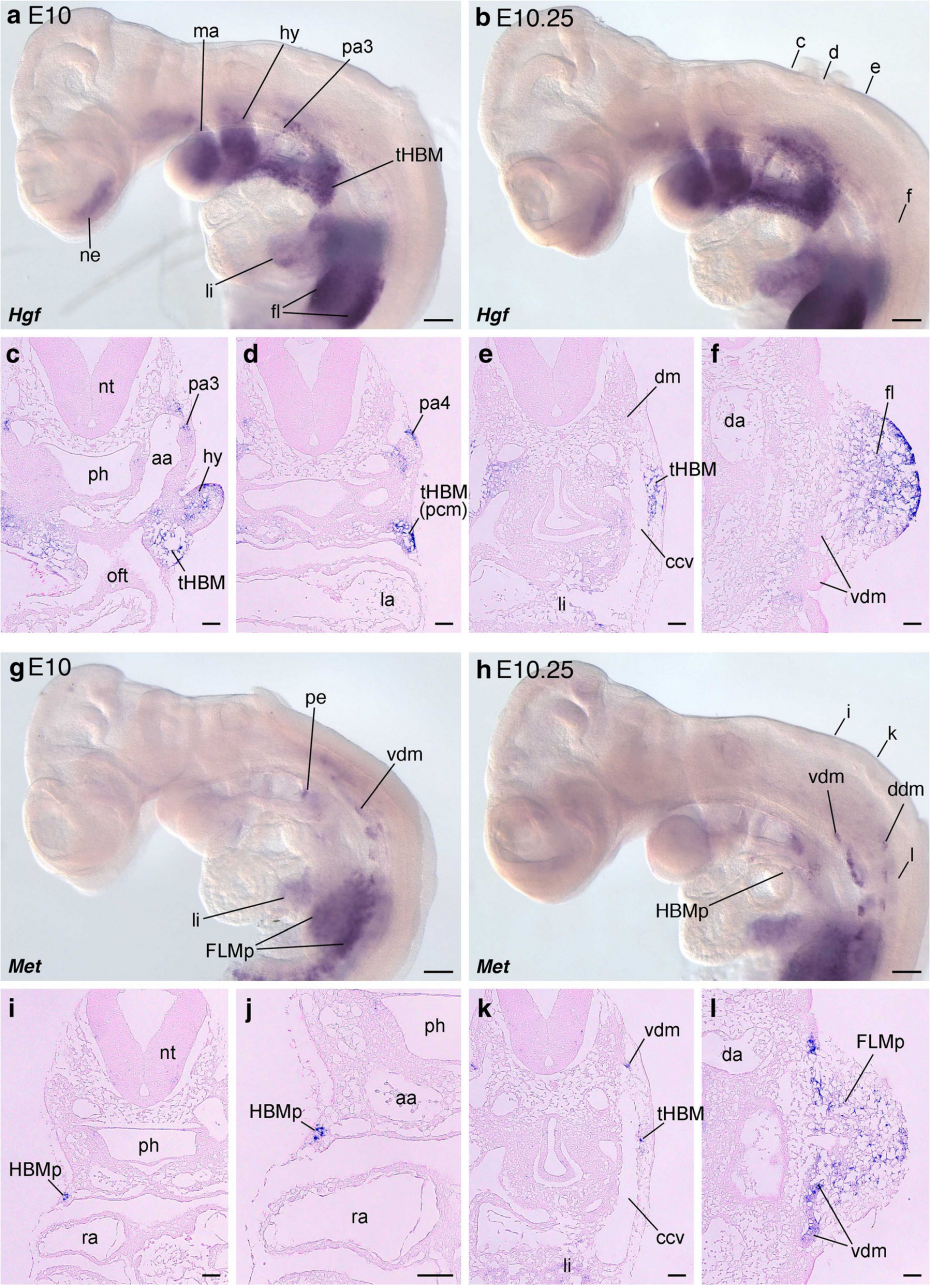
*Hgf* and *Met* expressions in mouse embryos. *Hgf* (**a**, **b**) and *Met* (**g**, **h**) expression pattern in mouse embryos at E10 (**a**, **c**) and E10.25 (**b**, **d**) stages. Lateral views. Transverse sections of E10.25 embryos after in situ hybridization with *Hgf* (**c**-**f**) and *Met* (**i**-**l**) probes. The dorsal pericardial region in (**i**) is magnified in (**j**). Section levels are indicated in (**b**) and (**h**). Note that *Hgf* is expressed in the migration route of HBM precursors, and also in the dorsal and ventral part of forelimb bud. *Met* expression was found in HBM and FLM precursors. aa, arch artery; ccv, common cardinal vein; da, dorsal aorta; ddm, dorsal part of dermomyotome; dm, dermomyotome; fl, forelimb bud; FLMp, forelimb muscle precursors; HBMp, hypobranchial muscle precursors; hy, hyoid arch; li, liver anlage; ma, mandibular arch; ne, nasal epithelium; nt, neural tube; oft, outflow tract; pa3, third pharyngeal arch; pa4, fourth pharyngeal arch; pe, pharyngeal endoderm; ph, pharynx; ra, right atrium; tHBM, trajectory of hypobranchial muscle; vdm, ventral part of dermomyotome. Scale bars on whole embryos, 200 μm. Scale bars on sections, 50 μm

*Hgf* transcripts were detected in the pharyngeal arches, the liver anlage, the dorsal and ventral portion of forelimb bud, and along the migration route of HBM precursors (tHBM) at E10, and a similar expression pattern was observed at E10.25, when HBM precursors circumvented the pharyngeal arches posteriorly (Fig. 1a-f) [33, 51]. In transverse sections after in situ hybridization, *Hgf* expression was detected in the central part of the hyoid arch and in the peripheral mesenchyme of pharyngeal arches (Fig. 1c, d). We also confirmed that *Hgf* was expressed in the mesenchyme lateral to the common cardinal vein, the dorsal part of the pericardium, and the medial part of the hyoid arch, in the region that HBM precursors pass through to reach the ventral pharynx (Fig. 1c-e) [51]. In addition, *Hgf* expression was detected in limb bud mesenchyme close to the ventrolateral lip of the dermomyotome (Fig. 1f).

At E10, *Met* mRNA was expressed weakly in pharyngeal endoderm and the liver anlage. Expression was also detected in the ventral part of the dermomyotome at the neck and limb bud levels and in FLM precursors in the limb bud (Fig. 1g). In E10.25 embryos, additional *Met* signals were detected in the dorsal parts of dermomyotome and HBM precursors (Fig. 1h). In transverse sections, we found *Met*-positive HBM precursor cells in the mesenchyme lateral to the common cardinal vein and the pericardial mesoderm, where *Hgf* expression was also detected (Fig. 1c-e, 1i-k). *Met* was also expressed in the ventrolateral lip of the dermomyotome and in delaminated FLM precursors in the limb bud (Fig. 1l).

Thus, our observations in mouse embryos revealed that *Met* is expressed in the ventral dermomyotome at the neck and forelimb levels and in the migrating HBM and FLM precursors, while *Hgf* is expressed in the mesenchyme near the ventral dermomyotome and along the migration route of HBM and FLM precursor cells.

### *Hgf* and *Met* expression and development of chick MMPs

We next examined *Hgf* and *Met* expressions in chick embryos, as no detailed analysis of *Met* gene expression during muscle development has been reported to date, and the spatiotemporal expression pattern of *Met* has not been compared thoroughly with that of *Hgf* in this model species [43, 47, 62, 63].

**Fig. 2:**
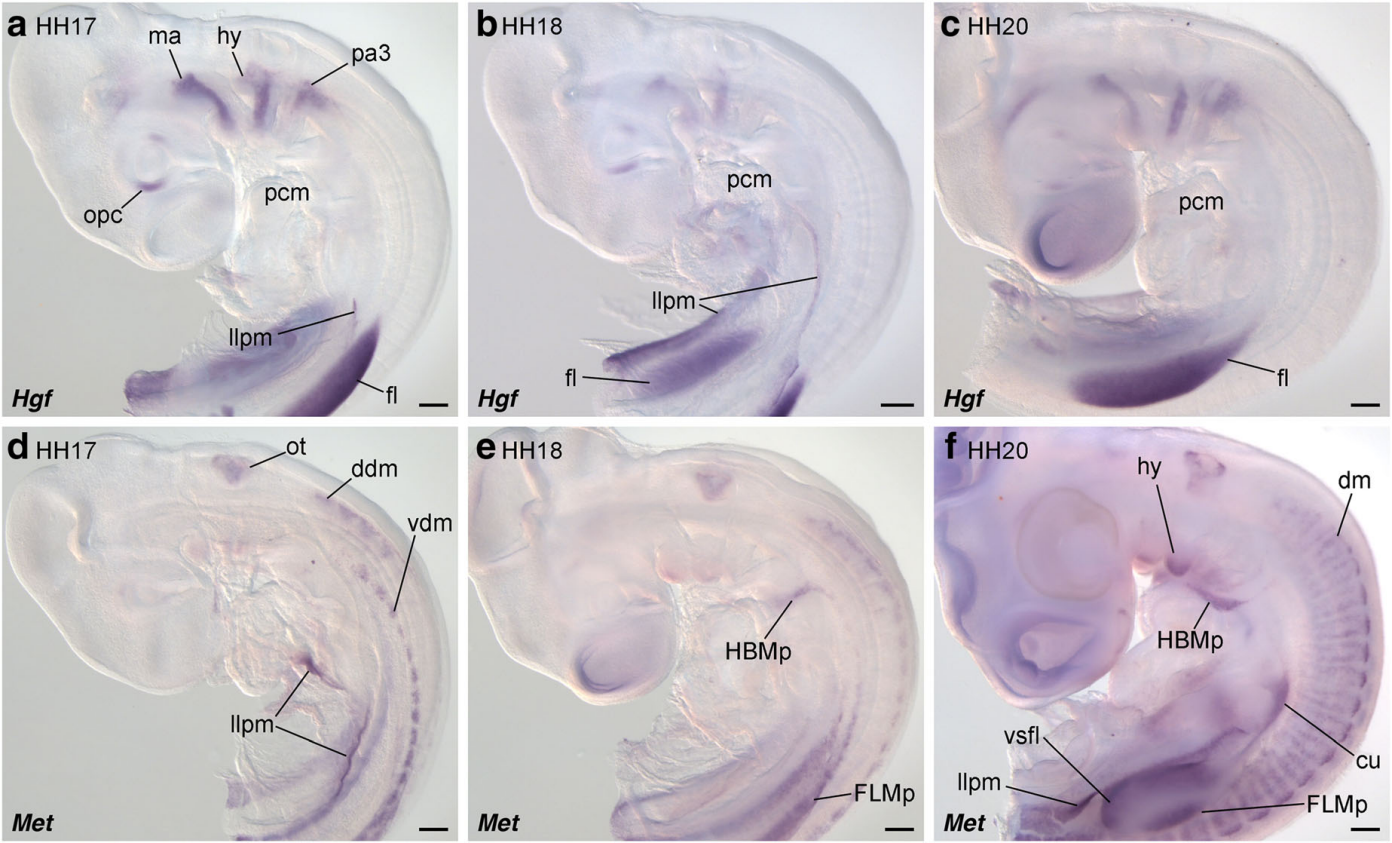
*Hgf* and *Met* expression patterns in chicken embryos. *Hgf* (**a**-**c**) and *Met* (**d**-**f**) expression pattern in chicken embryos at stage 17 (**a**, **d**), 18 (**b**, **e**) and 20 (**c**, **f**). Lateral views. *Hgf* is expressed in the whole forelimb bud, but not in the migration route of HBM precursors. *Met* expression was detected in the ventral part of dermomyotome and HBM precursors, and weakly in FLM precursors. cu, cucullaris muscle; ddm, dorsal part of dermomyotome; dm, dermomyotome; fl, forelimb bud; FLMp, forelimb muscle precursors; HBMp, hypobranchial muscle precursors; hy, hyoid arch; llpm, lateral part of lateral plate mesoderm; ma, mandibular arch; opc, optic cup; ot, otic vesicle; pa3, third pharyngeal arch; pcm, pericardium; vdm, ventral part of dermomyotome; vsfl, ventral surface of forelimb bud. Scale bars, 200 μm

At Hamburger and Hamilton stage 17 (HH17), when HBM precursors initiate delamination from the anterior somites, chicken *Hgf* was expressed in parts of the optic cup and in the mesoderm of the pharyngeal arches (Fig. 2a) [62, 64]. However, unlike in the mouse, *Hgf* transcripts were not detected along the trajectory of HBM precursors in chicken as reported previously (Fig. 2a) [64]. *Hgf* expression was observed uniformly in the limb buds and did not clearly exhibit dorsoventrally separated domains at stages HH17, HH18 or HH20 (Fig. 2a-c) [43, 47, 62]. *Hgf* expression was also found in the tissue lateral to the lateral plate mesoderm (Fig. 2a, b).

Chicken *Met* expression was detected in the otic vesicle, the dorsal and ventral portions of the dermomyotome, and the lateral tissue of the lateral plate mesoderm at stage HH17 embryos (Fig. 2d). At stage HH18, weak *Met* expression was observed in the precursor cells of the HBM and FLM (Fig. 2e). Chicken *Met* expression was sustained in HBM and FLM precursors at stage 20, and further detected in the ventral pharyngeal arches, cucullaris muscle progenitors, the anterior and posterior edges of the dermomyotome and in the ventral surfaces of limb buds (Fig. 2f) [65].

Thus, we found *Met* expressions in the ventral dermomyotome at the neck level and in HBM precursors, without obvious *Hgf* expressions in the neighboring tissues. This is in contrast to the situation in the mouse embryos where *Hgf* expression was also seen along the migration route of HBMs.

### *Hgf* and *Met* expression in shark embryos

**Fig. 3:**
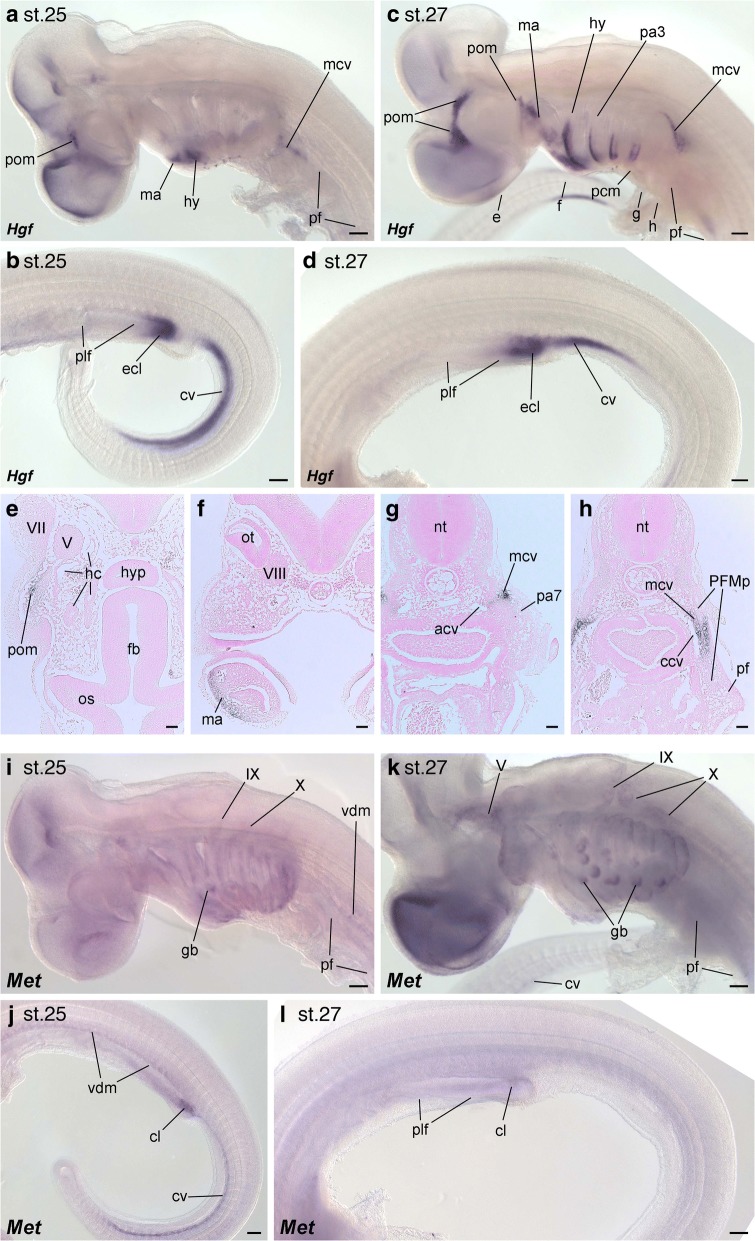
*Hgf* and *Met* expressions in shark embryos. *Hgf* (**a**–**h**) and *Met* (**i**–**l**) expression patterns in *S. torazame* embryos at stage 25 (**a**, **b**, **i** and **j**) and 27 (**c**–**h**, **k** and **l**). Lateral views of rostral (**a**, **c**, **i** and **k**) and caudal (**b**, **d**, **j** and **l**) parts of the embryos. Transverse sections of stage 27 embryos after in situ hybridization with *Hgf* probe (**e**–**h**). Section levels are indicated in (**c**). Shark *Hgf* is expressed in the posterior portion of fin buds, but not in the migration route of HBM precursors. *Met* expression was transiently detected in the ventral part of dermomyotome at stage 25, but not in the HBM and fin muscle precursors at stage 27. acv, anterior cardinal vein; ccv, common cardinal vein; cl, cloaca; cv, caudal vein; fb, forebrain; gb, gill buds; hc, head cavities; hy, hyoid arch; hyp, neurohypophysis; ma, mandibular arch; mcv, mesenchyme lateral to the cardinal vein; nt, neural tube; os, optic stalk; ot, otic vesicle; pa3, third pharyngeal arch; pa7, seventh pharyngeal arch; pcm, pericardium; pf, pectoral fin bud; PFMp, pectoral fin muscle precursors; plf, pelvic fin bud; pom, periocular mesenchyme; vdm, ventral dermomyotome; V, trigeminal nerve; VII, facial nerve; VIII, vestibulocochlear nerve; IX, glossopharyngeal nerve; X, vagus nerve. Scale bars on whole embryos, 200 μm. Scale bars on sections, 50 μm

The differential expression patterns of *Hgf* and *Met* between mouse and chicken embryos suggested further phylogenetic diversity of the patterns in these genes. To clarify the evolution of the HGF/MET signaling in MMPs, we performed a wider phylogenetic sampling of vertebrate taxa. Accordingly, we cloned *S. torazame Hgf* and *Met* genes, and performed in situ hybridization in developing shark embryos (Fig. 3 and Additional file 1 Figure S1-S3).

At stage 25, when HBM precursors start to extend ventrally from the anterior somites [51, 54], shark *Hgf* mRNA was detected in the periocular mesenchyme, pharyngeal arches and in the mesenchyme adjacent to the common cardinal vein (Fig. 3a). We also observed expression in the cloacal anlage and the possible caudal vein in the tail region, but *Hgf* transcripts were not observed in the pectoral or pelvic fin buds (Fig. 3a, b). At stage 27, when HBM precursors resided lateral to the pericardium and fin muscle precursors expanded into the fin bud mesenchyme [51], we found an *Hgf* expression pattern similar to that in stage 25 with additional *Hgf* signals in postotic pharyngeal arches and the posterior region of the pectoral fin buds (Fig. 3c-h). In histological sections, we confirmed *Hgf* expression in the ectomesenchyme of pharyngeal arches and the lateral mesenchyme of anterior and common cardinal veins (Fig. 3f-h). However, the pericardium, the anterior parts of pectoral fin buds and the pelvic fin buds were devoid of *Hgf* expression. A similar expression pattern was also observed at stage 28 (Additional file 1: Figure S3a), and we also found further expression domains of *Hgf* in the posterior part of the pelvic and dorsal fin buds (Additional file 1: Figure S3b, c).

Compared with *Hgf*, it was much more difficult to detect clear signals of *Met* in shark embryos, although we tried five distinct probes and two different protocols. A very faint, but relatively consistent expression pattern was observed in our in situ hybridization analyses with shark *Met* probe 1. We also performed in situ hybridization with the sense riboprobe of shark *Met* probe 1 (Fig. 3 and Additional file 1: Figure S3j, k). At stage 25, *Met* transcripts were detected in the cranial nerves (IX and X), the pharyngeal endoderm including gill buds and the ventral dermomyotome at the pectoral fin level (Fig. 3i). In the posterior part of the body, transcripts were observed in the ventral dermomyotome at inter-fin and pelvic fin levels, the cloacal anlage and the region of the caudal vein (Fig. 3j). At stage 27, *Met* expressions in the cranial nerves (V, IX, and X), gill buds, the cloaca anlage and the caudal vein were still detectable, but we did not find *Met* expression in the ventral part of the dermomyotome, nor in ventrally extending muscle precursors in the fin buds (Fig. 3k, l). At stage 28, we found *Met* expressions in the dorsal and posterior edges of the dermomyotome from postotic to pectoral fin levels, the posterior end of pectoral fin buds and the caudal part of intestinal primordium, including the cloacal anlage (Additional file 1: Figure S3d-g). Although in situ hybridization analysis could cause non-specific staining in tubular structures, such as gill buds and cloaca anlage, we constantly observed *Met* expression in those structures (Fig. 3i–l and Additional file 1: Figure S3d-g) (four of five embryos assayed at stage 25, all six embryos assayed at stage 27 and 11 of 12 embryos assayed at stage 28), and did not observe staining in embryos hybridized with *Met* sense probe (Additional file 1: Figure S3j, k) (two embryos). We occasionally observed *Met* expression in the dermomyotome regions at trunk and tail levels at stage 28 (four in twelve embryos), but expression was never detected in the muscle precursors of pelvic fins (Additional file 1: Figure S3f).

Overall, we did not detect *S. torazame Hgf* expression along the migration route of HBM precursors nor in the entire fin bud mesenchyme. In addition, we found no clear *Met* expression during the ventral extension of HBM and pectoral and pelvic fin muscle precursors in shark embryos.

### *Hgf* and *Met* expressions and lamprey HBM development

We did not observe *Hgf* and *Met* expression, as would be expected if these genes were involved in the delamination and migration of shark MMPs. However, these expression patterns could represent a derived condition of cartilaginous fishes, which exhibit an epithelial nature in various mesodermal tissues [1, 14, 66, 67]. To test this possibility, we assessed the expression patterns of *Hgf* and *Met* cognate genes during HBM development in the Arctic lamprey, which serves as an outgroup of gnathostomes for phylogenetic comparisons.

We screened a lamprey embryonic transcriptome based on RNA-sequencing data [68] and found two *Hgf*-like and two *Met*-like genes. Phylogenetic analyses revealed that the two *Hgf*-like genes were clustered outside the HGF and MST1 groups, so we named these two genes as *Hgf/Mst1A* and *Hgf/Mst1B*, respectively (Additional file 1: Figure S1). On the other hand, each of the two *Met*-like genes grouped with either MET or MST1R, suggesting—albeit not with strong bootstrap support—that the lamprey has a homolog for each gene (Additional file 1: Figure S2).

**Fig. 4:**
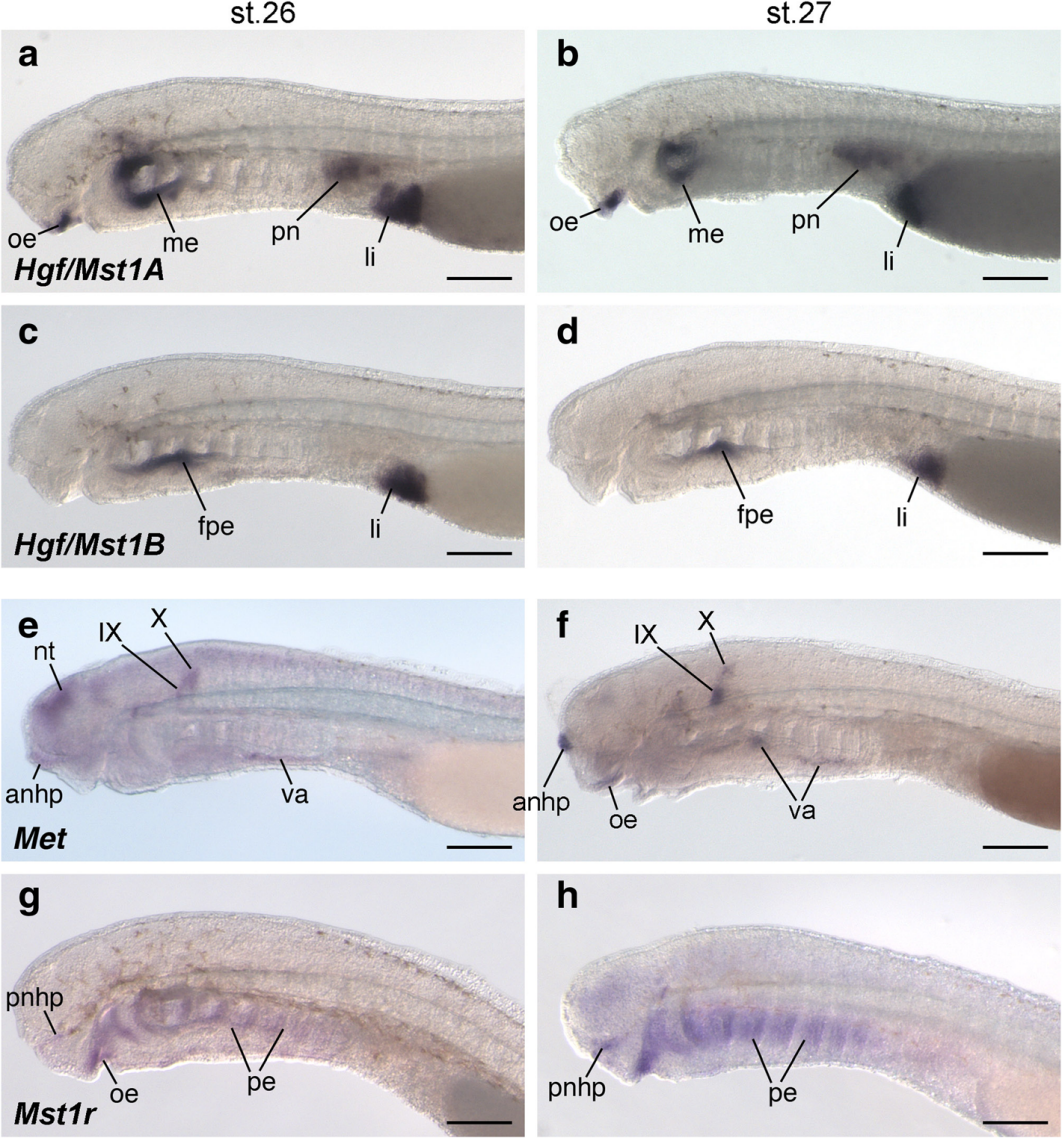
Expression pattern of *Hgf* and *Met* cognate genes in lamprey embryos. *Hgf/Mst1A* (**a**, **b**), *Hgf/Mst1B* (**c**, **d**), *Met* (**e**, **f**), *and Mst1r* (**g**, **h**) expression pattern in lamprey embryos at stage 26 (**a**, **c**, **e**, and **g**) and 27 (**b**, **d**, **f**, and **h**). Lateral views. Note that transcripts of *Hgf/Mst1A and B* genes were not detected in the tissue near the ventral part of dermomyotome, nor in the trajectory of HBM precursors. *Met* expressions were not observed in somites and HBM precursors. anhp, anterior nasohypophyseal placode; fpe, floor of pharyngeal endoderm; li, liver anlage; me, mandibular arch endoderm; nt, neural tube; oe, oral epithelium; pe, pharyngeal endoderm; pn, pronephros; pnhp, posterior nasohypophyseal placode; va, ventral aorta; IX, glossopharyngeal nerve; X, vagus nerve. Scale bars, 200 μm

We conducted in situ hybridizations of these four genes in stage 26 lamprey embryos, when the ventrolateral part of somites initiate the ventral extension above the pericardium, and at stage 27, when HBM precursors pass through the lateral side of the pericardium and posterior pharyngeal arches [51]. *Hgf/Mst1A* was expressed in the oral epithelium, the pharyngeal endoderm, pronephros and the liver anlage, and a similar expression pattern was observed in embryos at stage 27 (Fig. 4a, b). *Hgf/Mst1B* transcripts were detected in the floor of the pharyngeal endoderm and in the liver anlage at stages 26 and 27 (Fig. 4c, d). In addition to these stages, gene expression analyses were also performed in earlier and later stage embryos, yet neither gene was expressed along the trajectory of lamprey HBM (Additional file 1: Figure S4a, b).

Lamprey *Met* was expressed in the anterior portion of the nasohypophyseal placode, the brain and neural tube, the cranial nerves (IX and X) and the ventral aorta at stage 26. Later on, at stage 27, lamprey *Met* gained an additional expression domain in the oral epithelium, while it was downregulated in the central nervous system (Fig. 4e, f). In the meanwhile, *Mst1r* expression was detected in the posterior part of the nasohypophyseal placode, the oral epithelium and the pharyngeal endoderm during the same stages (Fig. 4g, h). However, expressions of *Met* and *Mst1r* in somites and HBM precursors were never observed in our analyses. We also investigated gene expression at other embryonic stages, but failed to detect the expression of either gene in the ventral dermomyotome or developing HBM precursors (Additional file 1: Figure S4c, d).

Thus, in contrast to the situation in mammals and birds, the receptor-ligand pair HGF/MET does not appear to be involved in the development of migratory myogenic progenitor cells of the HBM in lampreys.

## Discussion

A paracrine factor, HGF, and its receptor, MET, have been regarded as key signaling components regulating the development of MMPs in vertebrates. However, this idea is mainly based on results from mouse embryos, and a broader phylogenetic analysis has not been performed to date. Here, we investigated the embryonic expression patterns of *Hgf* and *Met* in the shark and lamprey, in addition to the mouse and chicken, and revealed that the spatiotemporal expression patterns of *Hgf* and *Met* were not consistent, but rather diverse among vertebrates during the development of HBMs and paired appendage muscles.

### HBM development and HGF/MET signaling

**Fig. 5:**
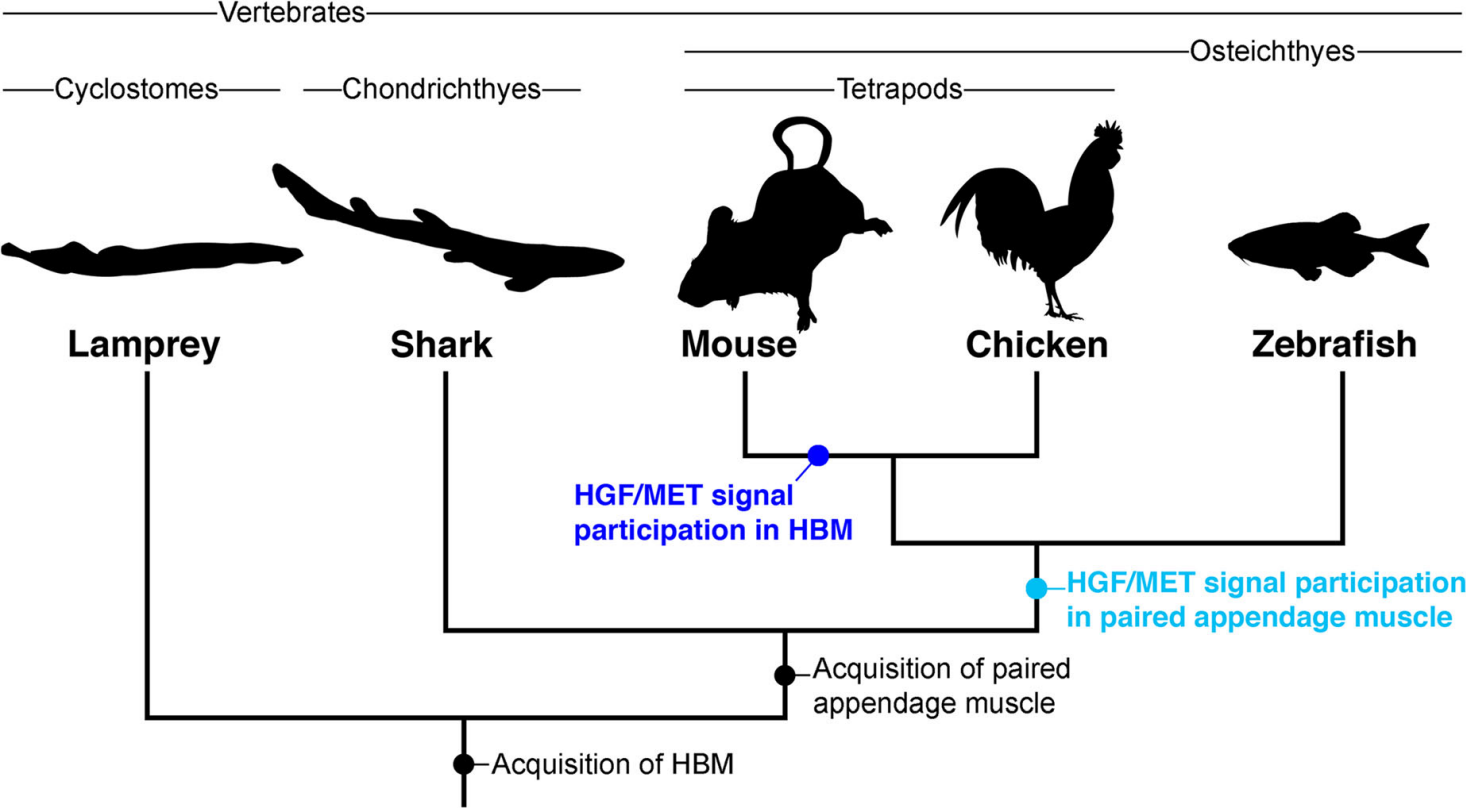
Stepwise participation of HGF/MET signaling in MMP development. A vertebrate phylogeny and a hypothetical scenario of the participation of HGF/MET signaling in MMPs. A stem osteichthyan acquired *Met* expressions in MMPs and *Hgf* expressions in fin buds, and established the delamination (and possibly the migration) of fin MMPs by HGF/MET signaling. A mouse ancestor gained *Hgf* expressions in the migration route of HBM and evolved HGF/MET signal pathway in HBM development. HBM, hypobranchial muscle

In mouse and chicken embryos, *Met* expression is initially observed in the ventral dermomyotome of anterior somites and subsequently in the ventrally extending HBM precursors (Figs. 1 and 2). A similar *Met* expression has also been found in zebrafish HBMs (sternohyoideus muscle) [45, 69], although not all studies reported this muscular expression [50, 70, 71]. *Hgf*, on the other hand, is expressed along the migration route of HBM precursors in the mouse (Fig. 1a–f), consistent with the function of HGF/MET signaling in the delamination and migration of the mouse HBM, as demonstrated previously [17, 33, 34, 36]. However, a similar spatial expression pattern of *Hgf* transcripts is not observed in chicken embryos (Fig. 2) [64], nor has it been reported in zebrafish [45, 70, 71]. In addition, shark and lamprey embryos did not show *Hgf* expression along the developmental trajectory of HBM, or *Met* expression in HBM precursors, either (Figs. 3 and 4). These data indicate that the expression patterns of *Hgf* and *Met* genes in HBM development are discordant among vertebrates, and that the regulation of HBM development by HGF/MET signaling may be an autapomorphic feature of mammals, or even possibly specific to the mouse lineage (Fig. 5).

As already mentioned, *Met* expression is observed in HBM precursors but *Hgf* transcripts do not accumulate along the migration route of the myogenic precursor cells in chicken and zebrafish embryos (Fig. 2) [45, 64, 69–71]. These observations imply an HGF-independent function of MET in HBM development [35, 72]. Alternatively, the expression of *Met* may be unrelated to the delamination and migration of HBM precursors, and mechanisms distinct from HGF/MET signaling may be involved in MMP development in these species [73]. Notably, our data suggest that other mechanisms must be involved in the migration of shark and lamprey HBMs. Even in the mouse embryo, the expression of *Met* seems to be detectable only partially in HBM precursors (Fig. 1) (compare with *Pax3* expression; [51]). Consistent with this, *Met* mutant mice show a defect only in a subset of HBMs (the tips of tongue muscles) [17]. These results suggest that additional mechanisms are also involved in HBM formation in the mouse.

### Limb/fin muscle development and HGF/MET signaling

We also found phylogenetically heterogeneous patterns of *Hgf* and *Met* expression in limb/fin muscle development. In mouse embryos, *Hgf* is expressed both in the dorsal and ventral parts of limb buds, while *Met* was expressed in the ventral dermomyotome and migrating FLM precursors (Fig. 1) [17, 38–41, 46, 48]. On the other hand, in chicken embryos, *Hgf* expression is detected in the whole limb bud mesenchyme at stages HH17–20 (Fig. 2), which has also been reported in earlier developmental stages [63]. At later stages, chick *Hgf* expression becomes restricted to the anterior part, and subsequently to the distal portions of limb buds [43, 47, 49]. Thus, unlike in the mouse, dorsoventrally separated *Hgf* domains are absent in chicken limb buds. As in the early stages of chicken *Hgf* expression patterns, zebrafish *hgfa* transcripts are observed in the entire fin buds before MMP migration and after the dorsoventral separation of pectoral fin muscle precursors [45, 50, 70, 71].

Despite the considerable differences in *Hgf* distribution in limb/fin buds, however, chicken and zebrafish *Met* genes are expressed in the ventral dermomyotome and delaminated myoblasts of paired appendages, as observed in mouse embryos (Fig. 2) [45, 50, 69, 71]. In contrast, we were unable to detect *Met* expression in fin muscle progenitors and *Hgf* expression in the whole fin buds in shark embryos (Fig. 3). Together with previous observations, these results suggest that the involvement of HGF/MET signaling in the delamination of limb/fin muscle progenitors is a trait present in the lineage of the Osteichthyes. Two alternative scenarios can be derived from these observations: either the involvement of HGF/MET signaling was an innovation in the lineage of bony fishes, and was not present in the last common ancestor of jawed vertebrates, or it was indeed present in the last common ancestor of gnathostomes, but was secondarily lost in chondrichthyans. Testing which scenario is correct would require studying other chondrichthyan embryos, such as the skate and elephant shark, in future research. However, considering the data at hand—for instance the lack of *Hgf* and *Met* expressions in MMP in the lamprey—we favor the former scenario in which HGF/MET signaling in MMPs of paired appendages was acquired in the lineage of bony fishes (Fig. 5). Last, the dorsoventral deployment of limb muscles by HGF/MET signaling seems to be an apomorphic trait of mammals (Fig. 5), but additional studies in other mammalian taxa will be necessary to characterize this mouse oddity.

The histological structure of fin myoblasts in cartilaginous fishes has long been a source of interest, and now it becomes a matter of debate whether it consists of epithelial progenitor cells specific to the lineage, or mesenchymal cells as in other gnathostomes [1, 14, 74, 75]. Because previous studies revealed the involvement of HGF/MET signaling in the delamination of the dermomyotome at the limb/fin levels in mouse, chicken and zebrafish embryos, and a recent study reported the de-epithelization of fin myoblasts in shark embryos [33, 42, 43, 45, 75], one would expect to detect *Hgf* and *Met* gene expression in shark fin buds. However, these genes were not expressed at the right time in the right place to allow them to be involved in the delamination and migration of paired fin myoblasts in shark embryos. Shark *Hgf* mRNA was detectable only in the posterior part of pectoral fin buds after the entrance of pectoral fin muscle precursors in the fin buds. Furthermore, shark *Met* was not expressed in pectoral and pelvic fin muscle precursors as they entered the fin buds (Figs. 3 and Additional file 1: Figure S3). Although further studies are needed, these data suggest that the early development of shark fin myoblasts is not regulated by HGF/MET signaling.

### Developmental nature of the vertebrate MMP

Understanding of vertebrate MMP development at the molecular level so far has included the expressions of *Pax3*, *Lbx1* and their related genes, together with the de-epithelialization and migration from the ventral dermomyotome controlled by HGF/MET signaling [12–16, 74–76]. However, in the present study, genetic control of these muscle development has diversified during evolution, while morphologically homologous patterns of the muscles have been largely conserved. This fact would provide an example of developmental system drift, in which developmental infrastructure can evolve without the pattern of phenotypes [77]: the MMP development appears to be independent from *Hgf* and *Met* expression both in lamprey and shark embryos, whereas both the genes are expressed in the muscular development of paired appendages in Osteichthyes, and finally the genes have become indispensable for the morphogenesis of mouse HBMs (Fig. 5). We thus propose a hypothesis that HGF/MET signaling participated in a stepwise manner in the MMP developmental program along vertebrate phylogeny (Fig. 5). Our study also unveiled MMP formation without *Hgf* and *Met* expression in the shark and lamprey, implying that the origin of the vertebrate MMP is independent of HGF/MET signaling. Further analyses of other ligands and receptors that are thought to play a role in MMP development and their genetic relationships with *Pax3* and *Lbx1* genes, would provide a better framework for the understanding of skeletal muscle development in vertebrates [13, 49, 78–81].

## Conclusions

Here, we used comparative embryological analyses to show that the expression patterns of *Hgf* and *Met* in MMP development are highly diverse across vertebrate species. In HBM development, we found both *Hgf* and *Met* expression only in mouse embryos. *Hgf* expression in limb/fin buds and *Met* expressions in limb/fin myoblasts were exclusively observed in Osteichthyan species. Furthermore, we found no evidence of the involvement of HGF/MET signaling in shark and lamprey MMP development. These observations demonstrate that HGF/MET signaling is not a general regulator of MMPs for all vertebrates, but rather suggest a stepwise participation of HGF/MET signaling in MMP development during evolution. Our results further suggest that other mechanisms are involved in MMP development, including in mouse HBMs.

## Additional file

Additional file 1: Supplementary Figures and Table. (DOCX 4908 kb)
