# Supplementary material for: Stepwise participation of HGF/MET signaling in the development of migratory muscle precursors during vertebrate evolution
Source: Zoological Lett. 2018 Jun 18;4:18. doi: 10.1186/s40851-018-0094-y (PMC6004694; doi:10.1186/s40851-018-0094-y)
Supplement: Supplementary file 1 — Supplementary Figures and Table. (DOCX 4908 kb) [file 40851_2018_94_MOESM1_ESM.docx]

**Additional file 1**

**Stepwise participation of HGF/MET signaling in the development of migratory muscle precursors during vertebrate evolution**

**Noritaka Adachi, Juan Pascual-Anaya, Tamami Hirai, Shinnosuke Higuchi, Shunya Kuroda and Shigeru Kuratani**

**Supplementary Figures
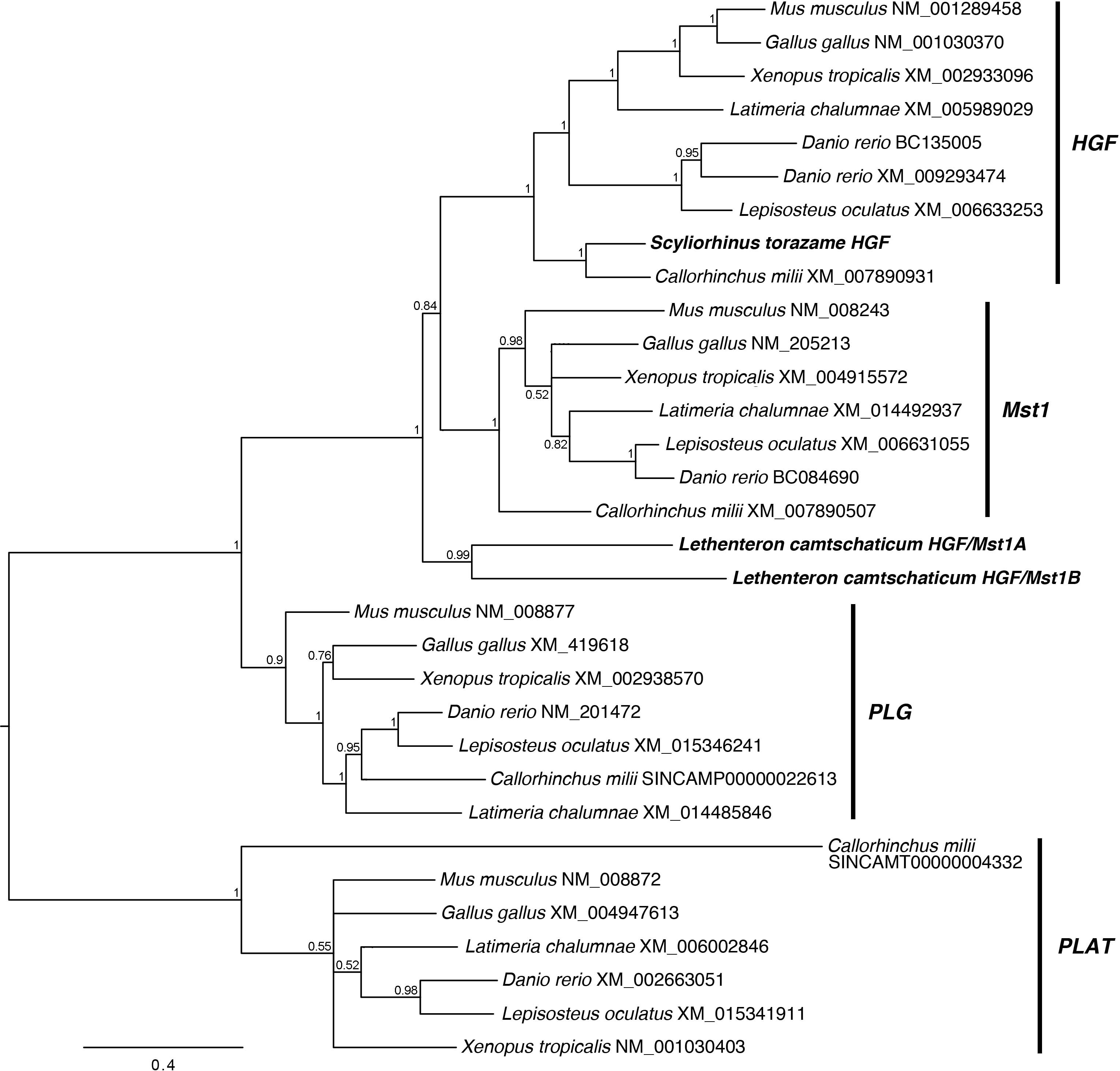
**

**Figure S1. Bayesian inference phylogenetic tree of HGF.** A 2,500,000-generation tree (1,875,000 post-burn trees) was constructed based on an alignment of 400 amino acid sites of HGF, MST1 (Macrophage stimulating 1), PLG (Plasminogen) and PLAT (Plasminogen activator, tissue), of which 357 were informative sites. Posterior probabilities above 0.5 are indicated. Legend at the bottom left indicates substitutions per site. *S. torazane* gene was clustered into HGF group. Two *L. camtschaticum* genes were placed outside of HGF and MST1 gene clusters, so we named them *Hgf/Mst1A* and *Hgf/Mst1B*, respectively. PLG and PLAT genes were used as outgroups. Accession numbers of sequences used in the analysis are indicated next to the corresponding species names. *S. torazane* and *L. camtschaticum* sequences were determined in this study and are indicated in bold.


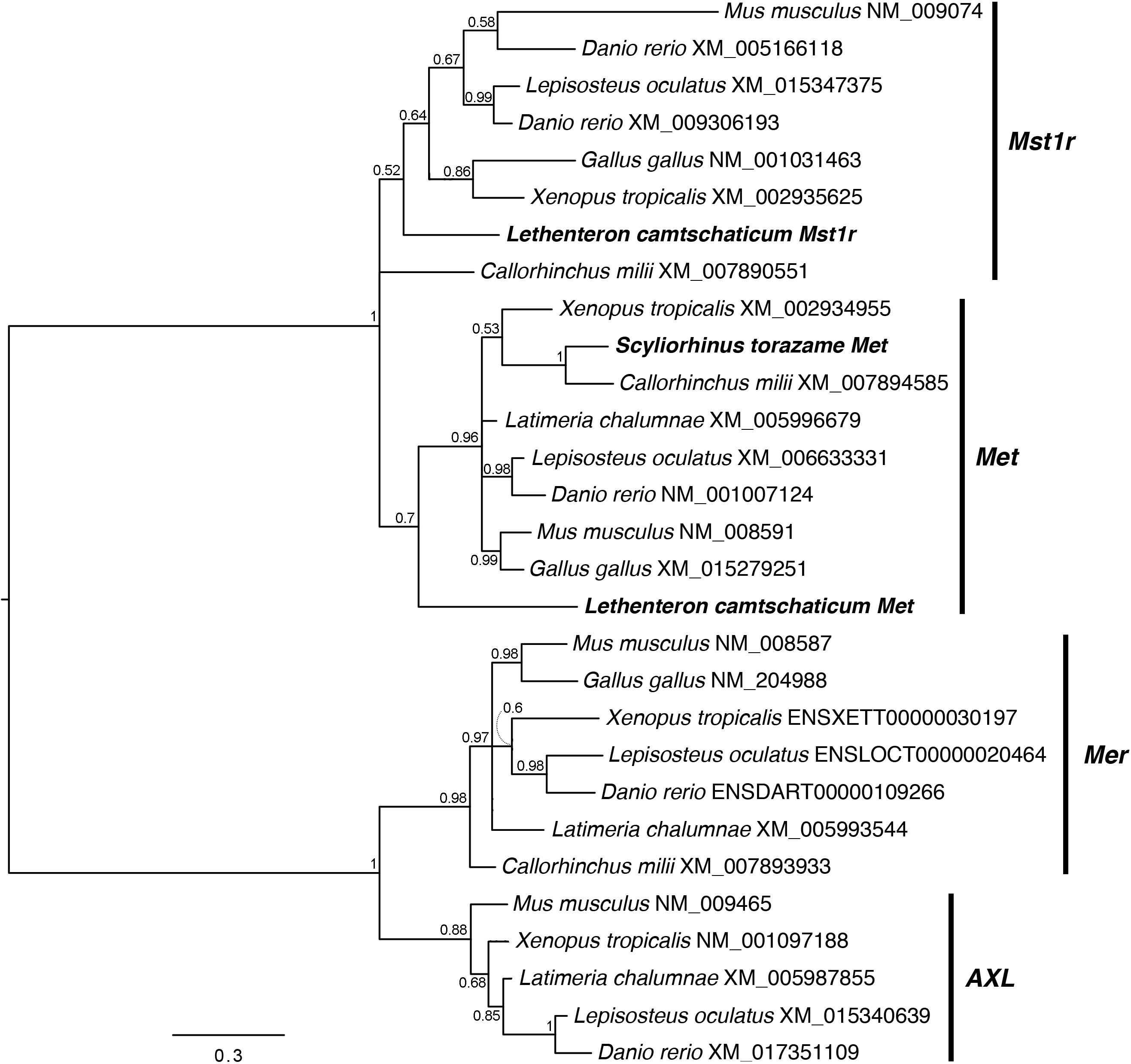


**Figure S2. Bayesian inference phylogenetic tree of MET.** A 2,500,000-generation tree (1,875,000 post-burn trees) of MET and related genes. An alignment of 137 amino acid sites of MET, MST1R (Macrophage stimulating 1 receptor), MER (C-mer proto-oncogene tyrosine kinase) and AXL (AXL receptor tyrosine kinase) with 120 informative sites, were used for the analysis. Posterior probabilities above 0.5 are indicated. Legend at the bottom left indicates substitutions per site. The shark gene and one of the lamprey genes were clustered within the MET group, while another lamprey gene was placed within the MST1R group. Note that the nodes including the lamprey genes are not highly supported. Accession numbers of sequences used in the analysis are indicated next to the corresponding species names. *S. torazane* and *L. camtschaticum* sequences were determined in this study and are indicated in bold. MER and AXL genes were used as outgroups.

**Figure S3.** ***Hgf* and *Met* expressions in shark embryos at stage 28.** Lateral views
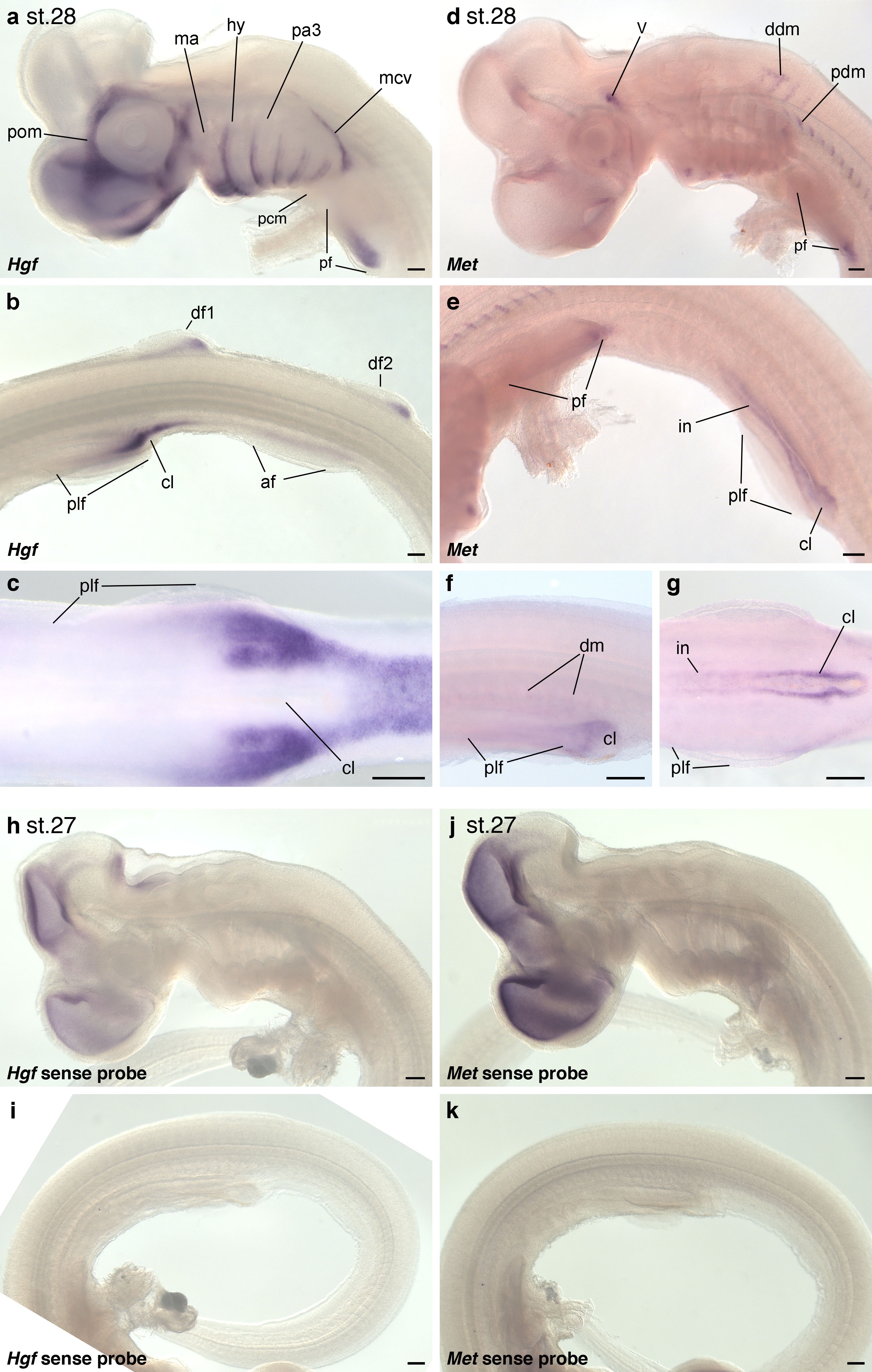
 (**a**, **b**, **d**-**f**, **h**-**k**) and ventral views (**c**, **g**). *S. torazame Hgf* expression was detected in the periocular mesenchyme, pharyngeal arches, the mesenchyme lateral to the cardinal vein, the mesenchyme around the cloaca and the posterior part of pectoral, pelvic and dorsal fin buds, but not in the pericardium and the anterior part of paired fin buds (**a**-**c**). Shark *Met* gene was expressed in the trigeminal nerve, the dorsal and posterior edges of dermomyotome, the posterior tip of pectoral fin buds and the posterior intestinal primordium including the cloaca (**d**-**g**). Occasionally weak *Met* expression was observed in the dermomyotome of trunk and tail levels, when we used the hybridization solution with dextran sulfate (**f**). *In situ* hybridization analyses with sense probes of shark *Hgf* and *Met* showed the background staining in the neural tube at stage 27 embryos (**g**-**j**). af, anal fin bud; cl, cloaca; ddm, dorsal part of dermomyotome; df1, first dorsal fin bud; df2, second dorsal fin bud; hy, hyoid arch; ma, mandibular arch; mcv, mesenchyme lateral to the cardinal vein; in, intestinal anlage; pa3, third pharyngeal arch; pcm, pericardium; pdm, posterior part of dermomyotome; pf, pectoral fin bud; plf, pelvic fin bud; pom, periocular mesenchyme; vdm, ventral part of dermomyotome; V, trigeminal nerve. Scale bars, 200 μm.


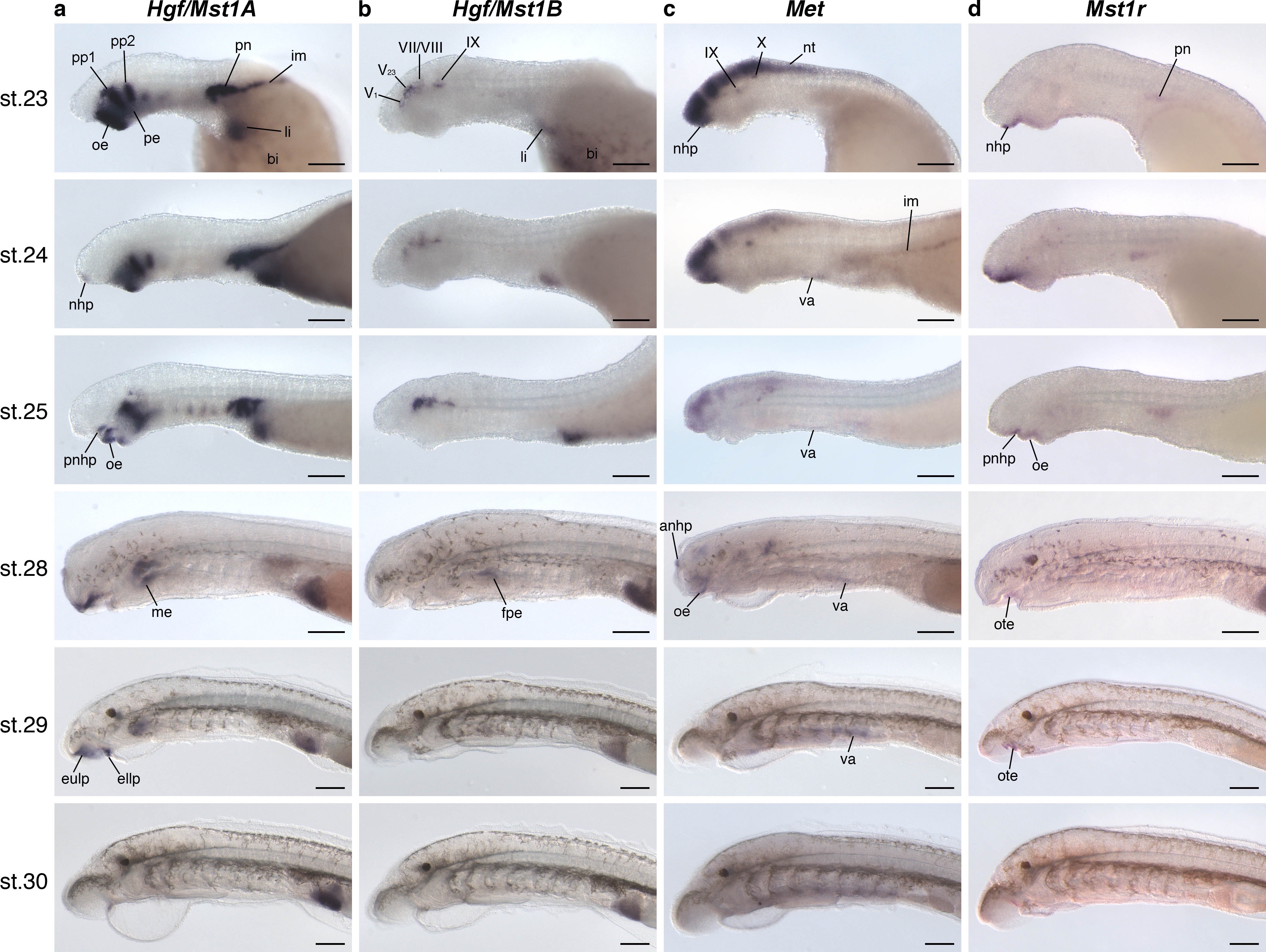


**Figure S4. Expression pattern of *Hgf* and *Met* cognate genes in lamprey embryos from stages 23 to 30.** *Hgf/Mst1A* signals were detected in the nasohypophyseal placode, oral epithelium and pharyngeal endoderm in the head region, and in the pronephros, intermediate mesoderm, liver anlage and possible bold islands in the trunk region (**a**). *Hgf/Mst1B* was expressed in the cranial nerve ganglia, pharyngeal endoderm, and liver anlage (**b**). *Met* expressions were observed in the neural tube, cranial nerve ganglia, nasohypophyseal placode, intermediate mesoderm and ventral aorta (**c**). *Mst1r* was expressed in the nasohypophyseal placode, pronephros, oral epithelium, pharyngeal endoderm, and oral tentacle (**d**). anhp, anterior nasohypophyseal placode; bi, blood islands; eulp, epithelium of upper lip; ellp, epithelium of lower lip; fpe, floor of pharyngeal endoderm; im, intermediate mesoderm; li, liver anlage; me, mandibular arch endoderm; nhp, nasohypophyseal placode; nt, neural tube; oe, oral epithelium; ote, oral tentacle; pe, pharyngeal endoderm; pn, pronephros; pnhp, posterior nasohypophyseal placode; pp1, first pharyngeal pouch; pp2, second pharyngeal pouch; va, ventral aorta; V1, ophthalmic nerve; V23, maxillomandibular nerve; VII/VIII, facial/vestibulocochlear nerve; IX, glossopharyngeal nerve; X, vagus nerve. Scale bars, 200 μm.


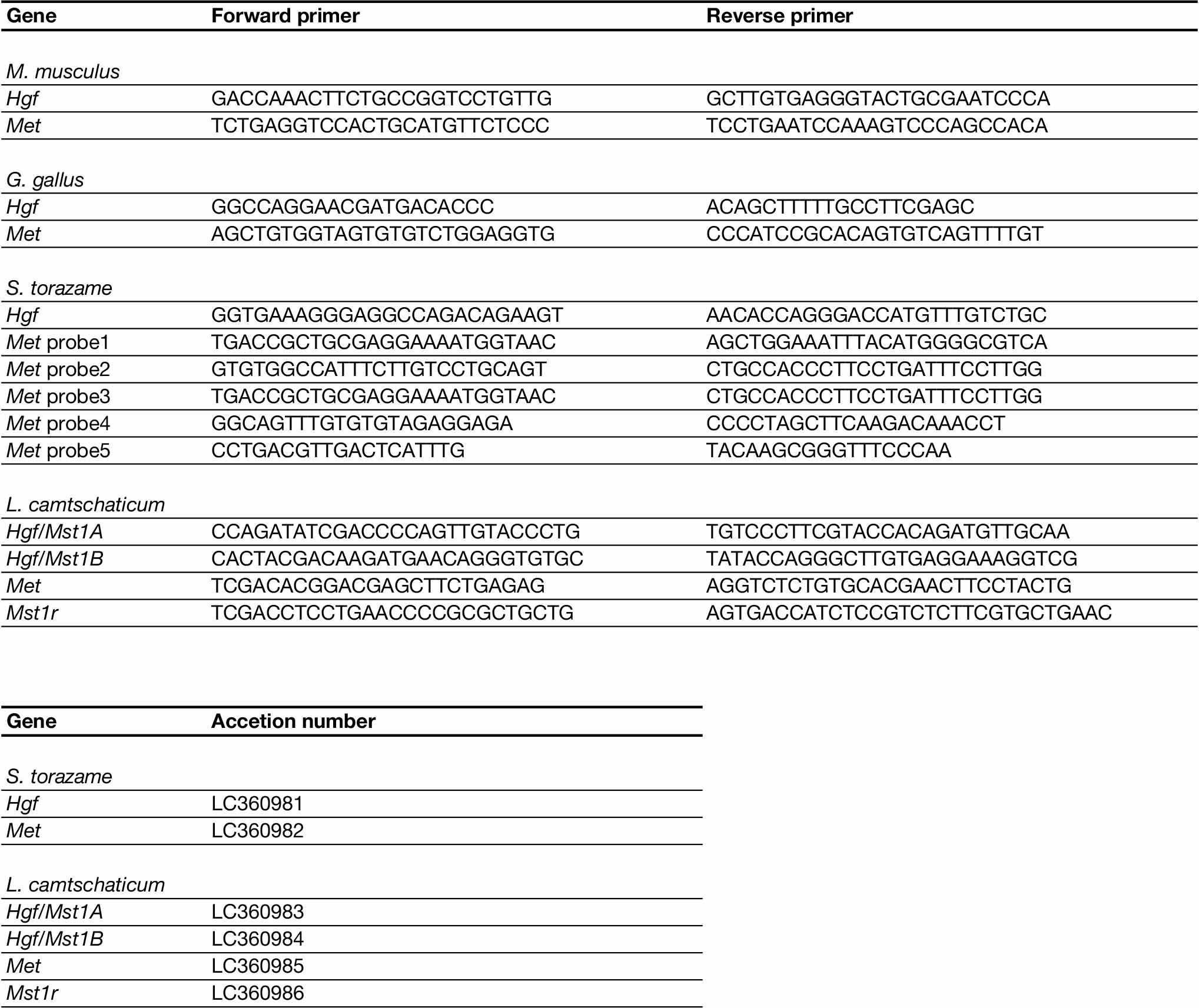


**Table S1.**
